# The Clinical Importance of 21-Day Combined Parenteral and Enteral Nutrition in Active Inflammatory Bowel Disease Patients

**DOI:** 10.3390/nu11092246

**Published:** 2019-09-18

**Authors:** Dorota Mańkowska-Wierzbicka, Jacek Karczewski, Ewelina Swora-Cwynar, Agnieszka Dobrowolska, Marta Stelmach-Mardas

**Affiliations:** 1 Department of Gastroenterology, Metabolic Diseases, Internal Medicine and Dietetics, Poznan University of Medical Sciences, Poznan 60-355, Poland; dmankowska.wierzbicka@gmail.com (D.M.-W.); jkarczewski@ump.edu.pl (J.K.); eswora@ump.edu.pl (E.S.-C.); adobzach@ump.edu.pl (A.D.); 2 Department of Environmental Medicine, Poznan University of Medical Sciences, Poznan 60-806, Poland; 3 Department of Biophysics, Poznan University of Medical Sciences, Poznan 60-780, Poland

**Keywords:** inflammatory bowel disease, enteral nutrition, parenteral nutrition, feeding

## Abstract

The aim of the study was to show the clinical magnitude of short-term feeding: enteral nutrition (EN) combined with parenteral nutrition (PN) in active Crohn’s disease and ulcerative colitis patients. Among 122 eligible inflammatory bowel disease (IBD) patients, 65 met the inclusion criteria. Combined EN and PN was administered for 21 days, wherein over the first 3–5 days of treatment, trophic enteral nutrition (300 kcal/day) was used with an energy increase of up to 1500 kcal. An EN was administered using a nasogastric tube or, in case of intolerance, using a naso-jejunal tube. For PN, the “All in One” system was used according to individually prepared admixtures (ESPEN Guidelines). In addition to routine blood measurement (i.e., ALAT, ASPAT, GGTP, creatinine, lipid profile), the following parameters were assessed: adiponectin, leptin, (hs)TNF-α, hsIL-6 and hsIL-10, TSH, NT-proBNP, serum vitamin B12 concentration, and tHcy. The results showed a considerable improvement in all clinically significant parameters (*p* < 0.05), showing the benefits and importance of short-term well-balanced EN combined with PN for nutritional and clinical status in IBD patients with active disease. The daily work at hospitals with active IBD patients demonstrates the potential of continued administration of home-based nutrition by patients.

## 1. Introduction

Several nutritional strategies have been used in patients suffering from inflammatory bowel disease (IBD). The management of malnutrition in IBD patients is related to increased requirements of energy and protein intake [1]. It has been shown that anti-inflammatory and low-fermentable oligosaccharide, disaccharide, monosaccharide (FODMAP), and polyol diets appear to be effective in reducing clinical symptoms and abdominal discomfort in IBD patients especially with inflammatory bowel syndrome (IBS) overlap [2]. Incorporation of components such as omega 3-fatty acids or glutamine may improve observed clinical symptoms; but, according to the European Society of Clinical Nutrition and Metabolism (ESPEN) guidelines, there are not enough data to dictate the use of such specific substrates in EN or PN in IBD patients [3]. Furthermore, the type of fat in enteral nutrition (EN) may be of high importance when it comes to inducing remission in active Crohn’s disease (CD) patients, whereas an excess of synthetic oleate in the enteral diet may preclude this effect can even be detrimental to these patients [4]. However, it is difficult to make patients comply with EN due to the mode of formula delivery, taste, and smell [5]. A recent publication of the ESPEN guideline by Forbes et al. [6] presents 64 recommendations on clinical nutrition in IBD patients including nine with very strong (Grade A) and 22 with strong recommendations (Grade B). One of them indicates that EN should always be preferred over the parenteral route, but combinations of EN and parenteral nutrition (PN) should be considered in patients for whom there is an indication for nutritional support and for whom > 60% of energy needs cannot be met via the enteral route (Grade A). There is limited literature on combined EN and PN run in prospectively controlled clinical trials with active patients, showing the need for further investigation [7]. 

Therefore, the aim of this study was to evaluate the effectiveness of short-term feeding with the use of combined EN and PN in active patients suffering from Crohn’s disease (CD) and ulcerative colitis (UC).

## 2. Materials and Methods

### 2.1. Study Design and Patients

This was a prospective clinically controlled intervention study. Written informed consent was obtained from all the participants prior to the study. Medical history and medications were recorded in an electronic database. The study protocol No. 42/09 was approved by the Research Ethical Committee of Poznan University of Medical Sciences in Poland and followed the requirements of the Declaration of Helsinki. 

One hundred twenty-two patients with IBD were screened for the study. Finally, 22 patients with active CD and 19 patients with active UC with mean age 34.0 (49% female and 51% male), mean body mass index (BMI) of 18.3 ± 2.1 kg/m^2^, and mean % fat mass (%FM) of 19.6 ± 4.2 were individually treated and fed at the Department of Gastroenterology at Poznan University of Medical Sciences. The study setting was an inpatient. In the study population, 42% of patients suffered from IBS overlap. Moreover, 24 patients were qualified for pharmacological treatment only (12 suffer from CD—control group, and 12 from UC—control group) with mean age 35.5 (50% men and 50% women), mean BMI: 18.28 ± 4.71, and mean % FM of: 20.01 ± 5.25. Ninety percent of the study population lived in a city with more than 500,000 of citizens and 1% were from the small cities (<5000 citizens). Additionally, 85% of them were married or lived with a partner. Moreover, 49% of individuals had secondary education, 5% higher education, 45% finished trade school, and 1% did not have education. More than 80% did not smoke. The control group did not receive nutritional intervention (a regular hospital diet). All patients were screened with the use of Nutritional Risk Score (NRS 2002) and the value exceeded 3 in all patients. Subjects enrolled in the study met the following inclusion criteria: moderately to severely active CD Activity Index CDAI > 450 and the Truelove–Witts Severity Index for UC with severe exacerbation Risk Score > 3 [6,7], body weight loss ≥ 5% in the last month, age >18, and willingness to participate in the study. Exclusion criteria were as follows: celiac disease, microscopic colitis, intestinal functional disorders, gastrointestinal cancer diseases, bacterial infections of the digestive tract (*Salmonellosis*, *Shigeliosis*, *Yersiniosis*, *Campylobacteriosis*), parasitic and viral diseases, drug or alcohol abuse, legal incompetence, and limited legal competence. 

### 2.2. Pharmacological Treatment, Enteral and Parenteral Nutrition

Standard pharmacological treatment was introduced in all patients: intravenous glucocorticoids—in the phase of acute exacerbation of the disease, patients received at the beginning Hydrocortizon 4 × 100 mg IV QDS for 4 to 5 days followed by orally administered Prednison: 40 mg/day (2/3 of the total dosage in the morning and 1/3 in the afternoon). Subsequently, based on the following gastrointestinal (GI) symptoms: ongoing and severe diarrhea (15 up to 25 watery stools) resulting in intestinal insufficiency with malabsorption, unintentional weight loss due to anorexia, nutritional deficiencies, dehydration, extreme tiredness, and severe malnutrition (chronic disease related malnutrition with inflammation based on BMI < 18.5 kg/m^2^ or weight loss (unintentional) > 10% indefinite of time, or > 5% over the last 3 months combined with either BMI < 20 kg/m^2^), patients were qualified for EN combined with PN to improve overall nutritional status and bowel insufficiency. Moreover, we noted the following adverse events: central line-associated bloodstream infections in three patients, diarrhea in four patients, and nausea and vomiting in two patients.

Combined EN and PN was administered after compensation for metabolic disorders. EN was administered using a nasogastric tube or, when intolerance proved to be an issue, using a naso-jejunal tube (45% of study patients). Due to the observed bowel insufficiency, a semielemental diet (1.0 kcal/mL) was applied with the use of a peristaltic pump and continuous infusion over 18–20 h/day to reduce the side effects. Over the first 3–5 days of treatment, trophic enteral nutrition (300 kcal/day) was used, and after stabilization of intestinal problems, the energy intake was increased to cover the requirement of each patients up to 1500 kcal. PN was conducted in the “All in One” system (AIO) according to individually prepared admixtures for each patient according to their requirements. Each admixture was prepared according to the standardized procedure by the Pharmacy of Heliodor Swiecicki Clinical Hospital at Poznan University of Medical Sciences. Following the ESPEN Guidelines [6], protein intake ranged between 1.2 and 1.5 g/kg/body mass/day and energy intake between 25 and 35 kcal/kg body mass/day. The nutritional composition of AIO was completed taking into account protein, fat (20% from MCT/LCT), carbohydrates (glucose), water- and fat-soluble vitamins, and trace elements. Additional enrichment with omega-3 fatty acids with a 10–20% ratio of selected fatty acids was used. To cover the requirements of energy and nutrients of a single patient, the reduction of PN was related to the introduction of enteral nutrition, where an EN was gradually replaced with an individually balanced diet. The combined EN and PN was administered by experienced medical staff and PN admixture composition was individually calculated by a physician and prepared by pharmacists. None of the patients failed to complete the study. Patients at home followed the FODMAP recommendations (only if needed) over a period of 6–8 weeks, gradually introducing previously eliminated foods to avoid abdominal discomfort. 

### 2.3. Anthropometry and Biochemical Assessment 

Body weight and body height approximated to 0.1 kg and 0.5 cm (Seca digital scale 763; Seca, Hamburg, Germany) were assessed to calculate BMI. The % of body fat mass was assessed with the use of Tanita MC 180 Multi Frequency Segmental Body Composition Analyser (Tanita MC 180, Tokyo, Japan). 

Biochemical assessment was performed in a certified laboratory according to standardized procedures and good laboratory practice at the baseline appointment and at the end of the study. Blood samples were taken after 14 hours of fasting. Total adiponectin and leptin concentrations were assessed using enzyme immunoassay methods (ALPCO, Diagnostics, Salem, NH, USA). Concentrations of human high sensitivity tumor necrosis factor α (hsTNF-α) were measured using ELISA test (eBioscience, San Diego, CA, USA), where the test sensitivity was at the level of 0.13 pg/mL; high sensitivity interleukin 6 (hsIL-6) and high sensitivity interleukin (hsIL-10) were measured by ELISA (eBioscience) with test sensitivity of 0.03 pg/mL and 0.05 pg/mL, respectively. Serum concentrations of high sensitivity tumor necrosis factor α (hsTNF-α), hsIL-6, and hsIL-10 were measured using ELISA test (eBioscience), with sensitivity levels of 0.13 pg/mL, 0.03 pg/mL, and 0.05 pg/mL, respectively.

Serum thyroid stimulating hormone (TSH) assay was performed using a third-generation electrochemiluminescence method (ECLIA), (Roche Diagnostics GmbH, Mannheim, Germany). The reference range was 0.27–4.2 μIU/mL. Concentrations of N-terminal prohormone of brain natriuretic peptide (NT-proBNP) was measured with the use of pro-BNP Elecsys cobas e100 test (Roche Diagnostics GmbH). The cutoff value for NT-proBNP was defined as <125 pg/mL. Serum vitamin B12 concentrations (pg/mL) were measured using ECLIA method tool on Cobas 6000 platform (Roche Diagnostics GmbH), and plasma homocysteine (tHcy) was measured by high-performance liquid chromatography (HPLC). The analyzed plasma Hcy compounds (Fluka Germany) were diluted with water at a 2:1 ratio and reduced using 1% TCEP (Tris-(2-carboxyethyl)-phosphin-hydrochloride; Applichem, Germany) at a 1:9 ratio. Subsequently, the samples were deproteinized using 1 M HClO_4_ (at a 2:1 ratio) and applied to the HPLC/EC system. The samples were fed to the HPLC system (P580A; Dionex, Idstein, Germany) coupled with an electrochemical detector (CoulArray 5600; ESA, North Chemsford, MA, USA). The analysis was performed in the Termo Hypersil Gold C18 column (250 mm × 4.6 mm × 5 µm) (Germany) in isocratic conditions, using a mobile phase of 0.15 M phosphate buffer, pH 2.9, supplemented with 12.5–17% acetonitrile for estimation of Hcy and 0.15 M phosphate buffer [8]. The system was controlled and the data were collected and processed using Chromeleon software (Dionex, Dreieich, Germany). The concentrations of serum total cholesterol (TC (mg/dL)), HDL-C (mg/dL), triglycerides (TG (mg/dL)), and glucose (mg/dL) were assessed with the use of enzymatic colorimetric methods (Diagnostics) and the activity of aspartate aminotransferase (AST) and alanine aminotransferase (ALT) using enzymatic methods. Serum creatinine was performed using the compensated (rate blanked) kinetic Jaffe method (Cobas 6000, Roche Diagnostics, Risch-Rotkreuz, Switzerland). Low-density lipoprotein-cholesterol (LDL-C (mg/dL)) was calculated according to the formula by Friedewald, Levy, and Fredrickson [9]. The basic, routine laboratory assessments were evaluated according to standardized procedure and monitored every 3 days. The recommendations of the National Health and Nutrition Examination Survey [10] and the American Diabetes Federation [11] were used for the results interpretation, respectively. 

### 2.4. Statistical Analysis

The data were presented as means ± SDs. The normality of the distribution was checked by the Shapiro–Wilk test. Comparisons between the groups were assessed using the Wilcoxon rank-sum test and the paired t-test (for data with normal distribution) to analyze the statistical differences between the variables before and after the intervention. Taking into account normality of the data for testing intergroup significance, either the Kruskal–Wallis test or one-way ANOVA with post-hoc test was used. The statistical significance level was 0.05. All calculations were performed using Statistica 10 software (TIBCO Software Inc., Palo Alto, CA, USA).

## 3. Results

The baseline clinical characteristics of the study population are presented in Table 1; Table 2. The patients suffering from CD and UC showed general clinical benefits from short-term combined EN and PN. In general, both CD and UC patients clinically benefitted from short-term combined EN and PN. Blood laboratory values were also significantly improved, taking into account the changes in the concentration of leukocytes, neutrocytes, fibrinogen, hemoglobin, hematocrit, erythrocytes, and platelet count (*p* < 0.05). The statistically significant changes in the concentration of selected biomarkers such as NT-proBNP, bilirubin, ALAT, and ASPAT during the combined EN and PN were beneficial in IBD patients (Table 3). The concentration of leptin significantly increased, whereas that of adiponectin significantly decreased. The level of NT-proBN decreased significantly (<125 pg/mL). Iron status showed statistical improvement and reached the recommended value of 37 µg/dL and above. Moreover, while vitamin B12 levels in the CD group statistically increased, homocysteine concentration for both groups with the nutritional support statistically decreased. Lipid profile (TC and LDL-cholesterol; TG in Ulcerative Colitis patients) changed significantly (Table 4). Similarly, there were also beneficial changes in electrolyte as well as CRP levels: we noted a 10-fold decrease in CRP concentration in CD patients and seven-fold decrease in UC patients. The concentrations of IL-6, IL-10, and TNF-α decreased significantly in both groups (Table 5). A statistically significant and clinically beneficial effect of combined nutrition was proven in intergroup analyses for leptin, NT-pro BNP, total protein, albumin, prealbumin, total cholesterol, HDL-cholesterol, LDL-cholesterol, triglycerides, potassium, calcium, magnesium, phosphates, CRP, and IL-6 (Table 1, Table 2, Table 3, Table 4 and Table 5). 

## 4. Discussion

The current study showed the benefits and importance of short-term enteral nutrition combined with parenteral nutrition as far as the clinical status of IBD patients with active disease is concerned.

Malnutrition in IBD patients is well documented and may be related to inadequate dietary intake, malabsorption, and disease activity [12]. It has been shown by Lochs et al. [13] that EN is less effective than a combination of 6-methylprednisolone and sulfasalazine in treating active Crohn’s disease patients, taking into account assessment of initial disease activity with the use of CDAI or disease location. In this context, we administered patients with standard pharmacological treatment, i.e., glucocorticosteroids and 5-ASA, in the initial phase and then incorporated the EN treatment. A study by Sakurai et al. [14] suggested that it is not necessary to restrict the amount of fatty acids (FA), specifically medium-chain triglycerides (MCTs), when given in liquid form to patients with active Crohn’s disease as both formulas with low and high content of MCTs induced clinical remission in about two-thirds of patients. Khosoo et al. [15] confirmed that a high-fat diet did not offer any nutritional advantage over a similar low-fat diet as the improvement in disease activity during feeding with a low-fat diet is comparable to that with a high-fat diet. Therefore, in the current study, 20% MCT/LCT was used in AIO and low-fat content, with 47% of the fat content from MCT used for easier fat absorption in EN. It also seems that nutritional supplementation with either an elemental or polymeric diet may provide a safe and effective alternative to chronic steroid therapy in patients with steroid-dependent Crohn’s disease, where polymeric and elemental diets are equally effective in active CD patients [16,17]. Rigaud et al. [18] pointed out that EN, whatever the diet, is an efficient primary therapy for active Crohn’s disease patients but does not influence the long-term outcome. However, González-Huix et al. [19] highlighted that total EN is safe and nutritionally effective, and in severe attacks of UC, it has been associated with fewer complications compared to PN. Therefore, taking into account the observed clinical manifestation of the disease, we have decided to follow recently published recommendations of ESPEN and combined EN and PN. As showed by Wright and Adler [20], the route of nutrient administration in acute exacerbation of regional enteritis does not appear to have any impact on the short-term outcome. Of course, liver and biliary abnormalities are well-known complications of IBD and thus alkaline phosphatase, serum bilirubin, aspartate aminotransferase, alanine aminotransferase, gamma-glutamyltransferase, lipid profile etc. are important biomarkers to be assessed during the feeding. The current study indicated improvement in these parameters. It seems that a more compromised nutrition and inflammation status with increased risk of cardiovascular diseases (CVD) can be observed in CD patients compared with UC patients [20]. However, in the current study, the concentration of NT-proBNP, recognized as a useful biomarker in the diagnosis of heart failure, decreased significantly in both patient groups (<125 pg/mL). Similarly, the concentration of CRP decreased significantly, i.e., 10-fold in CD patients and seven-fold in UC patients, confirming the importance of nutrition support in hospital environment. IL-6, IL-10, and TNF-α showed statistically significant decreases in the course of the patients’ feeding, which might be related to macrophage iron retention [21].

A higher prevalence of iron-deficiency anemia in addition to the presence of rheumatologic conditions, acid-related disorders, pain, bone diseases, migraines as well as cancer has been shown in IBD patients [22]. As shown by the German experience, the proportion of IBD patients with inadequately treated anemia/iron deficiency is unknown [23]. The gastroenterologists from nine European countries highlighted the need to increase the awareness and implementation of international guidelines on iron supplementation in IBD patients [24]. In general, in our study, the iron status during the combined EN+PN showed a statistical improvement and reached the recommended value of 37 µg/dL and above, showing another beneficial effect of such feeding. This is also a good prognosis for the future; however, the restriction in meat consumption commonly followed by IBD patients negatively influences their iron status [25]. Nevertheless, as shown the research by Vagianos et al. [26], food avoidance is common in patients with IBD and may be related to more personal preferences, showing the importance of nutritional education in that population to address food avoidance and introduce a balanced diet to prevent nutritional deficiencies. As showed by Yakut et al. [27], serum vitamin B12 deficiency is more common in CD patients compared to UC patients. Although in the current study the assessed serum concentration was in the reference range, statistical improvement was only observed in the CD group. As indicated by Battat et al. [28], true vitamin B12 deficiency is rare in IBD patients. However, it should be borne in mind that low vitamin B12 concentrations correlate with the concentration of homocysteine (high level-hyperhomocysteinemia) [29,30]. However, in the conducted study, a statistically significant decrease in homocysteine concentration (both CD and UC groups) was observed during the nutritional support, showing another beneficial effect of the feeding introduced by our team. On the contrary, Chen et al. [31] observed an increased plasma of homocysteine. Moreover, it was suggested that adipocytes may play a crucial role by actively participating in systemic immune responses in IBD patients, where leptin and adiponectin are associated with the disease severity and, similarly in our study, may show significant alterations of circulating serum levels of these adipokines in IBD patients [32,33]. For future monitoring of IBD activity, 78% of gastroenterologists from Switzerland considered clinical activity to be a more relevant criterion, whereas 15% preferred endoscopic severity and 7% preferred biomarkers [34]. Diarrhea is one of the most common symptoms in IBD, leading to changes in electrolyte transport being associated with intestinal inflammation [35]. Therefore, it is important to monitor electrolyte changes during the feeding, which in the case of our study were beneficial during EN+PN. 

All the procedures followed during the hospital stay for exacerbation of the disease should prepare every single patient for daily life. According to Cai et al. [36], apart from suffering from physical discomfort, diet and body image disturbances, and difficulties coping with activities of daily living, CD patients who self-administered nasogastric feeding faced many psychological challenges and needed physicians and nurses for assistance. Takagi et al. [37] indicated that at least the effectiveness of a “half elemental diet”, in which half of the daily energy requirement is provided by an elemental diet and the remaining half by a free diet, may be a promising maintenance therapy for CD patients. On the other hand, Yamamoto et al. [38] showed that concomitant EN during infliximab maintenance therapy does not significantly increase the maintenance rate of clinical remission in patients with CD. However, an EN therapy seems to reduce the incidence of postoperative CD recurrence [39]. There seems to be no consistent strategy at the different stages of disease manifestation. Patients, after such nutritional therapy and elimination of typical food allergens for a period of 21 days, may have difficulty in clinically tolerating a standard diet rich in fermentable carbohydrates [40,41]. The period of unloading the intestinal mucosa (PN and EN) from the standard diet probably also affects the intestinal microbiome [42]; therefore, during the transitional period, it is worth eliminating those elements of the diet that can potentially adversely affect the microbiota and thus translate into clinical ailments of the patient [43]. Moreover, it is well known that a low FODMAP diet may be an effective tool in the management of the common abdominal symptoms in patients with functional gastrointestinal symptoms once these molecules trigger these symptoms [44,45,46,47]. Thus, it may positively influence patient’s quality of life [48]. This diet may also reduce the expression of proinflammatory markers, such as C-reactive protein, and may interfere with the microbiome and its metabolites. The use of a low FODMAP diet can bring benefits to the IBD patients but may also modify their nutritional status [47]. Further research is required to determine the degree of FODMAP restriction required for symptom improvement [49].

## 5. Conclusions

Short-term combined EN and PN in active IBD patients seems to be of high clinical importance for future nutritional status management. The ESPEN Guideline should be followed at each stage of IBD treatment, while staying in line with good medical practice based on the clinical experience. 

## Figures and Tables

**Table 1:** Baseline characteristic of the study population (n = 65).

Analyzed Factor	Study Group		Control Group	
	CD (n = 22)	UC (n = 19)	CD (n = 12)	UC (n = 12)
**Age of diagnosis (years)**				
< 40	95.5	78.9	94.0	79.9
> 40	4.5	21.1	6.0	20.1
**Duration of disease (%)**				
< 1 year	22.3	42	13.6	15.8
1–5 years	45	36.82	31.8	21.1
> 5 years	32.7	21.2	9.1	26.3
**Previous surgery** (%)	22.0	0.0	9.1	0.0
**Crohn’s disease location (%)**				
ileum	18.2	-	4.5	-
colon	31.8	-	22.7	-
ileo-colon	45.5	-	27.2	-
upper GI	4.5	-	0.0	-
**Fistula occurrence (%)**				
perianal	18.2	-	2.0	-
subcutaneus	13.6	-	9.0	-
**Ulcerative colitis location (%)**				
Proctitis	-	0.0	-	0.0
left sided colitis	-	31.6	-	26.3
pancolitis	-	68.4	-	36.8
Medical treatment before study entry (%) *				
Aminosalicylates	99.9	100	100	100
Corticosteroids	54.5	52.6	75.0	66.7

* Aminosalicylates: mesalazine, sulfasalazine, corticosteroids budenoside, prednison, methylprednisolone.

**Table 2:** Complete blood count changes over combined parenteral and enteral nutrition in inflammatory bowel disease (IBD) patients (n = 65).

Analyzed Parameters	Group 1: Crohn’s Disease (n = 22)			Group 2: Ulcerative Colitis (n = 19)			Control Group Crohn’s Disease (n = 12)			Control Group Ulcerative Colitis (n = 12)			
	Baseline	Intervention	*p*-Value	Baseline	Intervention	*p*-Value	Baseline	Intervention	*p*-Value	Baseline	Intervention	*p*-Value	*p*-Value Inter Group
	Mean ± SD	Mean ± SD		Mean ± SD	Mean ± SD		Mean ± SD	Mean ± SD		Mean ± SD	Mean ± SD		
Leukocytes (10^3^/µL)	11.25 ± 6.61	7.68 ± 1.97	0.0156	11.06 ± 4.83	8.63 ± 2.69	0.1264	9.97 ± 4.26	9.02 ± 4.06	0.2439	10.59 ± 3.81	9.51 ± 3.87	0.2500	0.2439
Neutrocytes (10^3^/µL)	8.56 ± 4.59	5.71 ± 1.75	0.0195	15.88 ± 21.18	6.68 ± 3.10	0.0136	8.32 ± 3.88	7.48 ± 3.64	0.0024	8.35 ± 3.86	7.75 ± 3.26	0.2031	0.3259
Lymphocytes (10^3^/µL)	1.84 ± 1.64	1.37 ± 0.51	0.6553	2.71 ± 4.09	1.59 ± 0.67	0.4554	1.33 ± 0.79	1.47 ± 0.91	0.0266	1.40 ± 0.44	1.28 ± 0.41	0.9102	0.7815
Fibrinogen (mg/dL)	512.50 ± 153.50	334.40 ± 85.57	< 0.0001	538.90 ± 127.10	360.20 ± 80.09	< 0.0001	425.31 ± 59.33	405.93 ± 55.11	0.3054	396.00 ± 2.79	396.67 ± 93.30	0.8203	0.0527
Hemoglobin (g/dL)	9.66 ± 1.63	11.52 ± 1.83	0.0009	9.037 ± 1.97	10.76 ± 2.10	0.0227	11.67 ± 2.73	10.64 ± 2.76	0.0803	10.34 ± 2.16	10.19 ± 1.83	0.8201	0.3761
Hematocrit (%)	30.01 ± 4.61	34.33 ± 4.82	0.0172	28.51 ± 4.92	32.87 ± 4.89	0.0202	35.61 ± 7.27	32.53 ± 7.42	0.1465	31.18 ± 5.54	26.93 ± 9.28	0.4961	0.0724
Erythrocytes (10^6^/µL)	3.31 ± 0.77	4.08 ± 0.66	0.0015	3.14 ± 1.08	3.82 ± 0.54	0.0285	4.29 ± 0.87	3.89 ± 0.88	0.0866	3.67 ± 0.52	3.48 ± 0.59	0.0977	0.1577
Mean corpuscular volume (fL)	84.97 ± 8.41	83.82 ± 14.72	0.8233	84.86 ± 7.20	83.16 ± 13.98	0.7259	82.50 ± 6.73	83.48 ± 7.54	0.2253	85.66 ± 10.48	86.83 ± 9.95	0.1953	0.9061
Platelet count (10^3^/µL)	430.40 ± 143.00	377.20 ± 90.30	0.2648	593.60 ± 253.60	398.50 ± 129.10	0.0069	396.15 ± 133.61	349.79 ± 119.03	0.1855	432.11 ± 171.81	413.67 ± 200.01	0.4961	0.6332

**Table 3:** The changes during the combined enteral and parenteral nutrition in selected biomarkers in IBD patients (n = 65).

Analyzed Parameters	Group 1: Crohn’s Disease (n = 22)			Group 2: Ulcerative Colitis (n = 19)			Control Group Crohn’s Disease (n = 12)			Control Group Ulcerative Colitis (n = 12)			
	Baseline	Intervention	*p*-Value	Baseline	Intervention	*p*-Value	Baseline	Intervention	*p*-Value	Baseline	Intervention	*p*-Value	*p*-Value Inter Group
	Mean ± SD	Mean ± SD		Mean ± SD	Mean ± SD		Mean ± SD	Mean ± SD		Mean ± SD	Mean ± SD		
TSH (µU/mL)	2.29 ± 1.86	2.28 ± 0.44	0.0948	1.77 ± 1.00	1.98 ± 0.56	0.0171	1.86 ± 0.64	2.08 ± 0.59	0.1087	1.67 ± 0.30	1.83 ± 0.23	0.0078	0.0365
Leptin (pg/mL)	0.88 ± 1.19	2.97 ± 2.26	<0.0001	2.33 ± 6.34	4.14 ± 4.53	0.0005	1.85 ± 0.95	1.71 ± 0.93	0.3832	1.95 ± 0.70	2.17 ± 1.05	0.1484	0.0450
Adiponectin (µg/mL)	7.22 ± 2.16	4.35 ± 2.22	<0.0001	7.57 ± 4.21	4.81 ± 1.93	0.0111	5.10 ± 1.78	4.51 ± 1.91	0.0037	6.07 ± 2.32	5.72 ± 2.12	0.0273	0.3979
NT-proBNP (pg/mL)	289.10 ± 337.40	53.91 ± 32.97	0.0001	339.10 ± 623.80	62.37 ± 28.37	0.0015	391.61 ± 234.46	345.46.220.38	0.0012	318.33 ± 215.27	304.11 ± 232.27	0.5703	< 0.0001
Bilirubin (mg/dL)	0.29 ± 0.15	0.41 ± 0.19	0.0345	0.33 ± 0.21	0.44 ± 0.26	0.2609	0.38 ± 0.18	0.32 ± 0.18	0.3828	0.72 ± 0.75	0.60 ± 0.72	0.3125	0.5532
ALAT (U/L)	13.18 ± 9.68	22.59 ± 13.42	0.0019	25.79 ± 26.85	26.79 ± 7.28	0.0106	25.46 ± 22.97	22.61 ± 22.52	0.3013	28.33 ± 26.96	18.67 ± 14.25	0.1563	0.0415
ASPAT (U/L)	20.59 ± 17.40	22.05 ± 11.64	0.0953	18.26 ± 10.40	24.89 ± 7.45	0.0101	20.85 ± 12.83	22.07 ± 14.16	0.9697	18.78 ± 13.03	13.89 ± 6.92	0.1094	0.0140
GGTP (U/L)	47.77 ± 6.49	54.82 ± 47.06	0.0618	39.42 ± 13.76	45.16 ± 14.00	0.0794	45.46 ± 26.01	56.92 ± 46.24	0.7869	34.00 ± 22.08	25.33 ± 11.59	0.0977	0.0503
ALP (U/L)	129.60 ± 13.39	102.50 ± 6.93	0.3299	87.84 ± 36.17	99.89 ± 17.19	0.0847	98.23 ± 40.23	102.92 ± 30	0.5879	69.56 ± 27.90	68.44 ± 30.80	0.8203	0.0507
Urea (mg/dL)	18.95 ± 8.78	24.73 ± 6.94	0.0070	21.21 ± 6.93	25.84 ± 4.34	0.0216	26.46 ± 11.38	20.23 ± 6.98	0.0171	24.56 ± 8.38	18.56 ± 6.19	0.0078	0.0034
Creatinine (mg/dL)	0.65 ± 0.30	0.65 ± 0.20	0.6047	0.60 ± 0.21	0.75 ± 0.16	0.0141	0.83 ± 0.25	0.64 ± 0.24	0.0015	0.83 ± 0.21	0.71 ± 0.16	0.0273	0.2478
Uric Acid (mg/dL)	3.26 ± 1.11	3.01 ± 0.62	0.3654	2.77 ± 1.14	3.10 ± 0.55	0.1245	4.25 ± 1.27	3.85 ± 1.43	0.0005	4.62 ± 1.41	4.11 ± 1.07	0.0039	0.0059

TSH—thyroid-stimulating hormone; NT-proBNP—N-terminal pro-B-type natriuretic peptide; ALAT—alanine aminotransferase; ASPAT—aspartate transaminase; GGTP—gamma-glutamyl transpeptidase; ALP—alkaline phosphatase.

**Table 4:** The changes during the combined enteral and parenteral nutrition in selected nutritional parameters in IBD patients (n = 65).

Analyzed Parameters	Group 1: Crohn’s Disease (n = 22)			Group 2: Ulcerative Colitis (n = 19)			Control Group Crohn’s Disease (n = 12)			Control Group Ulcerative Colitis (n = 12)			
	Baseline	Intervention	*p*-Value	Baseline	Intervention	*p*-Value	Baseline	Intervention	*p*-Value	Baseline	Intervention	*p*-Value	*p*-Value Inter Group
	Mean ± SD	Mean ± SD		Mean ± SD	Mean ± SD		Mean ± SD	Mean ± SD		Mean ± SD	Mean ± SD		
Total protein (g/dL)	5.99 ± 1.08	6.90 ± 0.93	0.0119	5.14 ± 0.65	6.72 ± 0.45	< 0.0001	6.19 ± 1.22	5.52 ± 1.39	0.0002	6.40 ± 0.65	5.98 ± 0.86	0.0078	0.0054
Albumin (g/dL)	2.92 ± 0.67	3.74 ± 0.38	< 0.0001	2.63 ± 0.47	3.69 ± 0.36	< 0.0001	3.39 ± 0.89	3.00 ± 0.97	0.0002	3.63 ± 0.62	3.35 ± 0.65	0.0039	0.0028
Pre-albumin (g/L)	0.13 ± 0.07	0.34 ± 0.46	0.0005	0.13 ± 0.06	0.41 ± 0.77	< 0.0001	0.13 ± 0.03	0.12 ± 0.03	0.0001	0.14 ± 0.03	0.12 ± 0.02	0.0039	< 0.0001
Fe (µg/dL)	34.41 ± 19.42	56.73 ± 22.62	0.0002	24.21 ± 15.04	49.68 ± 21.34	< 0.0001	42.43 ± 24.82	50.77 ± 22.36	0.4332	36.22 ± 15.19	42.67 ± 22.57	0.0977	0.0640
TIBC (µg/dL)	241.2 ± 96.49	261.90 ± 92.00	0.3477	220.60 ± 95.30	264.60 ± 70.79	0.1700	209.69 ± 98.92	202.69 ± 92.67	0.7910	216.44 ± 52.65	208.11 ± 70.38	0.5703	0.0828
Vitamin B12 (pg/mL)	262.20 ± 129.40	391.70 ± 181.70	0.0036	441.50 ± 131.90	454.30 ± 153.10	0.8838	331.23 ± 191.73	318.08 ± 176.24	0.2163	378.00 ± 152.23	366.11 ± 137.41	0.2031	0.1554
Homocystein (µmol/L)	16.87 ± 16.79	7.54 ± 2.82	< 0.0001	10.92 ± 3.88	7.97 ± 3.72	0.0190	12.65 ± 1.74	11.53 ± 1.24	0.0134	12.60 ± 1.15	11.88 ± 1.10	0.0039	0.0005
Glucose (mg/dL)	87.36 ± 13.92	83.45 ± 7.94	0.5091	92.37 ± 16.84	86.16 ± 7.80	0.2136	91.23 ± 15.41	80.54 ± 10.51	0.0198	88.44 ± 12.24	90.22 ± 13.34	0.4258	0.2461
TC (mg/dL)	129.20 ± 40.02	157.40 ± 37.19	0.0195	130.90 ± 51.60	170.70 ± 30.25	0.0075	129.92 ± 40.17	126.00 ± 53.70	0.7354	139.00 ± 46.52	129.33 ± 32.97	0.8203	0.0018
HDL-C (mg/dL)	36.95 ± 16.18	46.68 ± 19.63	0.1156	40.68 ± 15.39	47.84 ± 15.22	0.3138	37.77 ± 20.59	35.00 ± 17.98	0.1272	39.89 ± 12.91	38.44 ± 10.09	0.4961	0.0914
LDL-C (mg/dL)	66.04 ± 31.03	85.32 ± 26.40	0.0120	72.56 ± 33.77	95.95 ± 27.22	0.0115	65.51 ± 28.11	69.23 ± 32.06	0.0574	61.82 ± 19.41	65.21 ± 20.42	0.2031	0.0274
TG (mg/dL)	104.70 ± 38.54	113.30 ± 39.59	0.5011	107.30 ± 36.07	139.50 ± 42.78	0.0136	104.62 ± 43.80	107.69 ± 40.86	0.6355	78.33 ± 22.52	78.22 ± 20.36	0.8203	0.0025
Sodium (mmol/L)	137.20 ± 3.13	140.90 ± 3.27	0.0006	129.00 ± 28.30	141.80 ± 1.58	< 0.0001	137.85 ± 5.89	141.46 ± 2.79	0.0244	138.67 ± 3.24	141.44 ± 4.19	0.0182	0.9314
Potassium (mmol/L)	3.97 ± 0.70	4.48 ± 0.28	0.0009	3.76 ± 0.73	4.42 ± 0.23	0.0002	4.14 ± 0.44	3.99 ± 0.64	0.3396	4.28 ± 0.44	3.91 ± 0.66	0.0469	0.0255
Calcium (mg/dL)	8.23 ± 1.08	9.21 ± 0.40	0.0002	8.56 ± 0.61	9.15 ± 0.48	0.0027	8.35 ± 0.76	8.32 ± 0.48	0.6377	8.30 ± 1.49	8.24 ± 1.38	0.6470	< 0.0001
Magnesium (mg/dL)	2.02 ± 0.27	3.54 ± 4.37	< 0.0001	2.06 ± 0.20	2.66 ± 0.38	< 0.0001	2.17 ± 0.63	2.04 ± 0.53	0.1514	2.06 ± 0.34	1.83 ± 0.31	0.0005	< 0.0001
Phosphates (mg/dL)	3.41 ± 0.70	7.60 ± 10.84	< 0.0001	3.02 ± 0.66	4.08 ± 0.48	< 0.0001	2.80 ± 0.75	2.73 ± 0.84	0.4131	2.71 ± 0.57	2.31 ± 0.44	0.0039	< 0.0001

TC—total cholesterol; LDL—cholesterol—low density lipoprotein; HDL—cholesterol—high density lipoprotein; TG—triglycerides.

**Table 5:** The changes during the combined enteral and parenteral nutrition in selected inflammatory parameters in IBD patients (n = 65).

Analyzed Parameters	Group 1: Crohn’s Disease (n = 22)			Group 2: Ulcerative Colitis (n = 19)			Control Group Crohn’s Disease (n = 12)			Control Group Ulcerative Colitis (n = 12)			
	Baseline	Intervention	*p*-Value	Baseline	Intervention	*p*-Value	Baseline	Intervention	*p*-Value	Baseline	Intervention	*p*-Value	*p*-Value Inter Group
	Mean ± SD	Mean ± SD		Mean ± SD	Mean ± SD		Mean ± SD	Mean ± SD		Mean ± SD	Mean ± SD		
hsCRP (mg/L)	80.23 ± 84.16	10.84 ± 8.78	<0.0001	67.94 ± 64.34	9.78 ± 5.00	< 0.0001	68.44 ± 55.52	36.29 ± 32.83	0.0022	58.28 ± 41.78	45.24 ± 44.40	0.2500	< 0.0001
ESR (mm/h)	49.82 ± 35.19	22.00 ± 19.01	0.0034	66.89 ± 32.37	16.42 ± 9.16	< 0.0001	32.38 ± 19.20	28.07 ± 21.95	0.2134	39.89 ± 30.25	38.78 ± 26.56	0.1953	0.1860
Procalcytonin (ng/mL)	0.25 ± 0.29	0.05 ± 0.02	0.0014	0.12 ± 0.16	0.05 ± 0.01	0.2852	0.25 ± 0.61	0.11 ± 0.13	0.3375	0.22 ± 0.20	0.17 ± 0.10	0.1250	0.7424
hsTNF-α (pg/mL)	0.13 ± 0.04	0.08 ± 0.05	0.0349	0.27 ± 0.59	0.10 ± 0.05	0.0113	0.10 ± 0.05	0.09 ± 0.05	0.8995	0.08 ± 0.06	0.07 ± 0.05	0.9988	0.3334
IL-6 (pg/mL)	7.83 ± 2.95	4.45 ± 2.20	0.0010	8.97 ± 2.63	5.73 ± 2.47	0.0001	8.13 ± 2.98	7.77 ± 2.60	0.0713	7.78 ± 2.74	7.82 ± 2.71	0.5703	0.0012
IL-10 (pg/mL)	4.44 ± 6.63	2.21 ± 2.87	0.2569	6.73 ± 6.20	3.65 ± 2.64	0.0769	4.10 ± 2.49	3.16 ± 1.99	0.0871	4.60 ± 3.50	4.16 ± 3.00	0.3008	0.0778

hsCRP—high sensitivity C-reactive protein; ESR—erythrocyte sedimentation rate; hsTNF-α—tumor necrosis factor-α high sensitivity; IL-6—interleukin-6; IL-10—interleukin-10.

## References

[1] Sökülmez P., Demirbağ A.E., Arslan P., Dişibeyaz S. (2014). Effects of enteral nutritional support on malnourished patients with inflammatory bowel disease by subjective global assessment. Turk J. Gastroenterol..

[2] Yoon S.R., Lee J.H., Lee J.H., Na G.Y., Lee K.H., Lee Y.B., Jung G.H., Kim O.Y. (2015). Low-FODMAP formula improves diarrhea and nutritional status in hospitalized patients receiving enteral nutrition: A randomized, multicenter, double-blind clinical trial. Nutr. J..

[3] Sugihara K., Morhardt T.L., Kamada N. (2019). The Role of Dietary Nutrients in Inflammatory Bowel Disease. Front. Immunol..

[4] Gassull M.A., Fernández-Bañares F., Cabré E., Papo M., Giaffer M.H., Sánchez-Lombraña J.L., Richart C., Malchow H., González-Huix F., Esteve M. (2002). Eurpoean Group on Enteral Nutrition in Crohn’s Disease. Fat composition may be a clue to explain the primary therapeutic effect of enteral nutrition in Crohn’s disease: Results of a double blind randomised multicenter European trial. Gut.

[5] Yamamoto T., Shimoyama T., Kuriyama M. (2017). Dietary and enteral interventions for Crohn’s disease. Curr. Opin. Biotechnol..

[6] Forbes A., Escher J., Hébuterne X., Kłęk S., Krznaric Z., Schneider S., Shamir R., Stardelova K., Wierdsma N., Wiskin A.E. (2017). ESPEN guideline: Clinical nutrition in inflammatory bowel disease. Clin. Nutr..

[7] Abad-Lacruz A., Gonzalez-Huix F., Esteve M., Fernández-Bañares F., Cabré E., Boix J., Acero D., Humbert P., Gassull M.A. (1990). Liver Function Tests Abnormalities in Patients with Inflammatory Bowel Disease Receiving Artificial Nutrition: A Prospective Randomized Study of Total Enteral Nutrition vs Total Parenteral Nutrition. J. Parenter. Enter. Nutr..

[8] Accinni R., Bartesaghi S., De Leo G., Cursano C.F., Achilli G., Loaldi A., Cellerino C., Parodi O. (2000). Screening of homocysteine from newborn blood spots by high-performance liquid chromatography with coulometric array detection. J. Chromatogr. A.

[9] Friedewald W.T., Levy R.I., Fredrickson D.S. (1972). Estimation of the concentration of low-density lipoprotein cholesterol in plasma, without use of the preparative ultracentrifuge. Clin. Chem..

[10] Ford E.S., Mokdad A.H., Giles W.H., Mensah G.A. (2003). Serum total cholesterol concentrations and awareness, treatment, and control of hypercholesterolemia among US adults: Findings from the National Health and Nutrition Examination Survey, 1999 to 2000. Circulation.

[11] American Diabetes Association (2014). Diagnosis and classification of diabetes mellitus. Diabetes Care.

[12] Vagianos K., Bector S., McConnell J., Bernstein C.N. (2007). Nutrition Assessment of Patients with Inflammatory Bowel Disease. J. Parenter. Enter. Nutr..

[13] Lochs H., Steinhardt H.J., Klaus-Wentz B., Zeitz M., Vogelsang H., Sommer H., Fleig W.E., Bauer P., Schirrmeister J., Malchow H. (1991). Comparison of enteral nutrition and drug treatment in active Crohn’s disease: results of the European Cooperative Crohn’s Disease Study IV. Gastroenterology.

[14] Sakurai T., Matsui T., Yao T., Takagi Y., Hirai F., Aoyagi K., Okada M. (2002). Short-term efficacy of enteral nutrition in the treatment of active Crohn’s disease: A randomized, controlled trial comparing nutrient formulas. J. Parenter. Enter. Nutr..

[15] Khoshoo V., Reifen R., Neuman M.G., Griffiths A., Pencharz P.B. (1996). Effect of Low- and High-Fat, Peptide-Based Diets on Body Composition and Disease Activity in Adolescents with Active Crohn’s Disease. J. Parenter. Enter. Nutr..

[16] Verma S., Holdsworth C.D., Giaffer M.H. (2001). Does Adjuvant Nutritional Support Diminish Steroid Dependency in Crohn Disease?. Scand. J. Gastroenterol..

[17] Verma S., Brown S., Kirkwood B., Giaffer M. (2000). Polymeric versus elemental diet as primary treatment in active Crohn’s disease: A randomized, double-blind trial. Am. J. Gastroenterol..

[18] Rigaud D., Cosnes J., Le Quintrec Y., Rene E., Gendre J.P., Mignon M. (1991). Controlled trial comparing two types of enteral nutrition in treatment of active Crohn’s disease: Elemental versus polymeric diet. Gut.

[19] González-Huix F., Fernández-Bañares F., Esteve-Comas M., Abad-Lacruz A., Cabré E., Acero D., Figa M., Guilera M., Humbert P., de León R. (1993). Enteral versus parenteral nutrition as adjunct therapy in acute ulcerative colitis. Am. J. Gastroenterol..

[20] Wright R.A., Adler E.C. (1990). Peripheral parenteral nutrition is no better than enteral nutrition in acute exacerbation of Crohn’s disease: A prospective trial. J. Clin. Gastroenterol..

[21] Cavallaro F., Duca L., Pisani L.F., Rigolini R., Spina L., Tontini G.E., Munizio N., Costa E., Cappellini M.D., Vecchi M. (2017). Anti-TNF-Mediated Modulation of Prohepcidin Improves Iron Availability in Inflammatory Bowel Disease, in an IL-6-Mediated Fashion. Can. J. Gastroenterol. Hepatol..

[22] Bähler C., Schoepfer A.M., Vavricka S.R., Brüngger B., Reich O. (2017). Chronic comorbidities associated with inflammatory bowel disease: Prevalence and impact on healthcare costs in Switzerland. Eur. J. Gastroenterol. Hepatol..

[23] Blumenstein I., Dignass A., Vollmer S., Klemm W., Weber-Mangal S., Stein J. (2014). Current practice in the diagnosis and management of IBD-associated anaemia and iron deficiency in Germany: The German AnaemIBD Study. J. Crohn’s Colitis.

[24] Stein J., Bager P., Befrits R., Gasche C., Gudehus M., Lerebours E., Magro F., Mearin F., Mitchell D., Oldenburg B. (2013). Anaemia management in patients with inflammatory bowel disease: Routine practice across nine European countries. Eur. J. Gastroenterol. Hepatol..

[25] Vidarsdottir J.B., Johannsdottir S.E., Thorsdottir I., Bjornsson E., Ramel A. (2016). A cross-sectional study on nutrient intake and -status in inflammatory bowel disease patients. Nutr. J..

[26] Vagianos K., Clara I., Carr R., Graff L.A., Walker J.R., Targownik L.E., Lix L.M., Rogala L., Miller N., Bernstein C.N. (2016). What Are Adults with Inflammatory Bowel Disease (IBD) Eating? A Closer Look at the Dietary Habits of a Population-Based Canadian IBD Cohort. JPEN J. Parenter. Enter. Nutr..

[27] Yakut M., Üstün Y., Kabacam G., Soykan I. (2010). Serum vitamin B_12_ and folate status in patients with inflammatory bowel diseases. Eur. J. Intern. Med..

[28] Battat R., Kopylov U., Byer J., Sewitch M.J., Rahme E., Nedjar H., Zelikovic E., Dionne S., Bessissow T., Afif W. (2017). Vitamin B_12_ deficiency in inflammatory bowel disease: A prospective observational pilot study. Eur. J. Gastroenterol. Hepatol..

[29] Mahmood A., Needham J., Prosser J., Mainwaring J., Trebble T., Mahy G., Ramage J. (2005). Prevalence of hyperhomocysteinaemia, activated protein C resistance and prothrombin gene mutation in inflammatory bowel disease. Eur. J. Gastroenterol. Hepatol..

[30] Romagnuolo J., Fedorak R.N., Dias V.C., Bamforth F., Teltscher M. (2001). Hyperhomocysteinemia and inflammatory bowel disease: Prevalence and predictors in a cross-sectional study. Am. J. Gastroenterol..

[31] Chen M., Mei Q., Xu J., Lu C., Fang H., Liu X. (2012). Detection of melatonin and homocysteine simultaneously in ulcerative colitis. Clin. Chim. Acta.

[32] Morshedzadeh N., Rahimlou M., Asadzadeh Aghdaei H., Shahrokh S., Reza Zali M., Mirmiran P. (2017). Association Between Adipokines Levels with Inflammatory Bowel Disease (IBD): Systematic Reviews. Dig. Dis. Sci..

[33] Karmiris K., Koutroubakis I.E., Kouroumalis E.A. (2008). Leptin, adiponectin, resistin, and ghrelin—Implications for inflammatory bowel disease. Mol. Nutr. Food Res..

[34] Schoepfer A.M., Vavricka S., Zahnd-Straumann N., Straumann A., Beglinger C. (2012). Monitoring inflammatory bowel disease activity: Clinical activity is judged to be more relevant than endoscopic severity or biomarkers. J. Crohn’s Colitis.

[35] Sullivan S., Alex P., Dassopoulos T., Zachos N.C., Iacobuzio-Donahue C., Donowitz M., Brant S.R., Cuffari C., Harris M.L., Datta L.W. (2009). Down-Regulation of Sodium Transporters and NHERF Proteins in IBD Patients and Mouse Colitis Models: Potential Contributors to IBD-associated Diarrhea. Inflamm. Bowel Dis..

[36] Cai Q., Li F., Zhou Y. (2018). Experiences of Chinese patients with Crohn’s disease in the self-administration of nasogastric feeding: A descriptive qualitative study. PLoS ONE.

[37] Takagi S., Utsunomiya K., Kuriyama S., Yokoyama H., Iwabuchi M., Takahashi H., Takahashi S., Kinouchi Y., Hiwatashi N., Funayama Y. (2006). Effectiveness of an ‘half elemental diet’ as maintenance therapy for Crohn’s disease: A randomized-controlled trial. Aliment. Pharmacol. Ther..

[38] Yamamoto T., Nakahigashi M., Umegae S., Matsumoto K. (2010). Prospective clinical trial: Enteral nutrition during maintenance infliximab in Crohn’s disease. J. Gastroenterol..

[39] Yamamoto T., Shiraki M., Nakahigashi M., Umegae S., Matsumoto K. (2013). Enteral nutrition to suppress postoperative Crohn’s disease recurrence: A five-year prospective cohort study. Int. J. Colorectal Dis..

[40] Chandler M. (2013). Focus on nutrition: Dietary management of gastrointestinal disease. Compendium.

[41] Nie Y., Lin Q., Luo F. (2017). Effects of Non-Starch Polysaccharides on Inflammatory Bowel Disease. Int. J. Mol. Sci..

[42] Basson A., Trotter A., Rodriguez-Palacios A., Cominelli F. (2016). Mucosal Interactions between Genetics, Diet, and Microbiome in Inflammatory Bowel Disease. Front. Immunol..

[43] Hou J.K., Lee D., Lewis J. (2014). Diet and Inflammatory Bowel Disease: Review of Patient-Targeted Recommendations. Clin. Gastroenterol. Hepatol..

[44] Ruemmele F.M. (2016). Role of Diet in Inflammatory Bowel Disease. Ann. Nutr. Metab..

[45] Pedersen N., Ankersen D.V., Felding M., Wachmann H., Végh Z., Molzen L., Burisch J., Andersen J.R., Munkholm P. (2017). Low-FODMAP diet reduces irritable bowel symptoms in patients with inflammatory bowel disease. World J. Gastroenterol..

[46] Kakodkar S., Mutlu E.A. (2017). Diet as a therapeutic option for adult inflammatory bowel disease. Gastroenterol. Clin. N. Am..

[47] Barbalho S.M., Goulart R.D.A., Aranão A.L.D.C., De Oliveira P.G.C. (2018). Inflammatory Bowel Diseases and Fermentable Oligosaccharides, Disaccharides, Monosaccharides, and Polyols: An Overview. J. Med. Food.

[48] Elhusseiny M.H., Amine A.K.E., Salem O.I., Tayel D.A., Elsayed E. (2018). Low FODMAP diet in Egyptian patients with Crohn’s disease in remission phase with functional gastrointestinal symptoms. JGH Open.

[49] Whelan K., Cox S.R., Prince A.C., Myers C.E., Irving P.M., Lindsay J.O., Lomer M.C. (2017). Fermentable Carbohydrates [FODMAPs] Exacerbate Functional Gastrointestinal Symptoms in Patients with Inflammatory Bowel Disease: A Randomised, Double-blind, Placebo-controlled, Cross-over, Re-challenge Trial. J. Crohn’s Coliti.

